# P-2045. Riding the Storm: Leveraging a Best Practice Advisory to Mitigate IV Shortages Post-Hurricane

**DOI:** 10.1093/ofid/ofaf695.2209

**Published:** 2026-01-11

**Authors:** Rebecca Y Linfield, Sean Carlton, David J Epstein, Amy Chang, David Svec, Jonathan H Chen, Lisa Shieh

**Affiliations:** Stanford University, Stanford, California; Stanford Health Care, Stanford, California; Stanford Medicine, Stanford, CA; Stanford University, Stanford, California; Stanford University, Stanford, California; Stanford University, Stanford, California; Stanford University, Stanford, California

## Abstract

**Background:**

In September 2024, Hurricane Helene severely damaged Baxter International’s North Carolina manufacturing facility, leading to severe intravenous (IV) fluid shortages. Stanford Medicine implemented warnings in the electronic medical record (EMR) to shift providers away from IV medications. We sought to understand whether there was increased acceptance of the pre-existing Best Practice Advisory (BPA) with additional notifications in the EMR.

**Figure 1:**
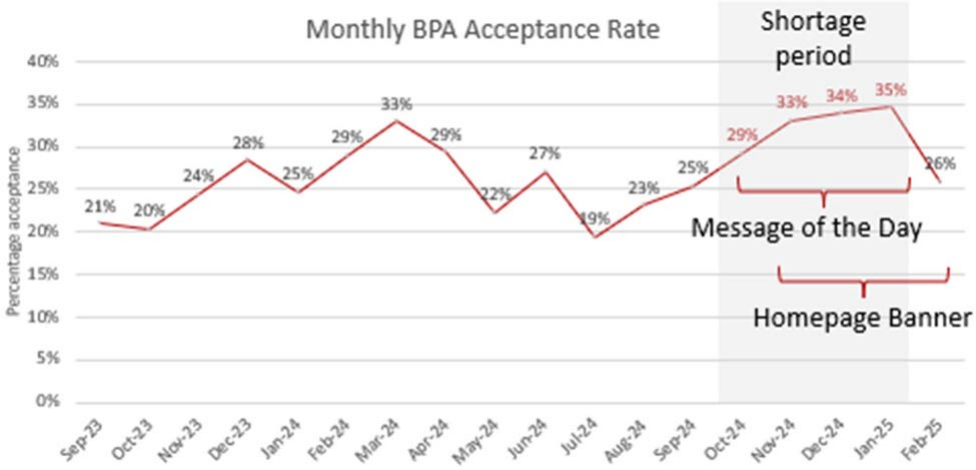
Monthly BPA Acceptance Rate

**Table 1:**
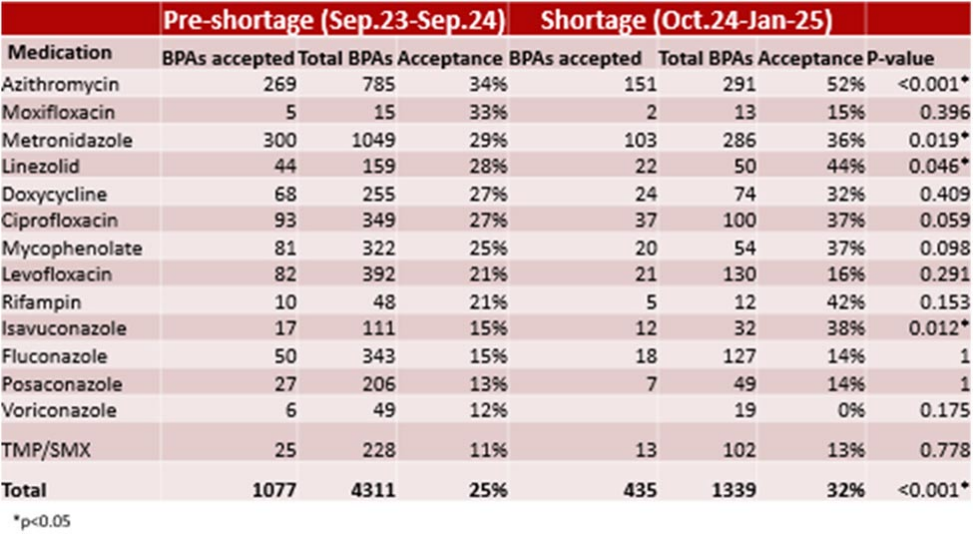
Acceptance Rates by Medication Pre-Shortage and Shortage

**Methods:**

We analyzed BPA acceptance rates from September 2023 through February 2025 for

Stanford Hospital and Stanford Tri-Valley Hospital. The EMR Epic displayed additional notifications about the IV fluid shortage as: (a) Message of the Day at sign-in from Oct. 11, 2024 through Jan. 15, 2025 and (b) Homepage Banner in Summary Report from Nov. 21, 2024 through Feb. 25, 2025. A Chi-square test or Fisher’s Test was used to determine statistical differences in the pre-shortage and shortage periods.

**Results:**

Overall BPA acceptance increased from an average of 25% pre-shortage to 32% during the IV fluid shortage (p< 0.001). The increase in BPA acceptance was driven mostly by antibiotics and not antifungals, with the the biggest changes observed for azithromycin (from 34% acceptance rate to 52%, p< 0.001), metronidazole (from 29% to 36%, p = 0.019), linezolid (from 28% to 44%, p = 0.046), and isavuconazole (from 15% to 38%, p = 0.012), which are often used in oral formulation in more stable patients. The BPA acceptance decreased to baseline in February 2025 at 26% acceptance rate when the Message of the Day was taken down, suggesting it is more effective than the Homepage Banner.

**Conclusion:**

Additional EMR notifications were associated with increased acceptance of the BPA shifting IV to oral medications. Future studies must evaluate how to maximize this benefit while minimizing alert fatigue.

**Disclosures:**

Jonathan H. Chen, MD, PhD, Reaction Explorer: Ownership Interest

